# Psychiatrist treatment choices in schizophrenia according to clinical vignettes

**DOI:** 10.1192/j.eurpsy.2026.10282

**Published:** 2026-07-31

**Authors:** M. Rojnic Kuzman

**Affiliations:** 1 Zagreb school of medicine; 2 Zagreb University Hospital Centre, Zagreb, Croatia

## Abstract

**Abstract:**

Based on the 2023 Ambassador study on schizophrenia, conducted among 454 psychiatrists and trainees from 35 European countries using six clinical vignettes (cases A–F), we aimed to explore therapeutic approaches across different phases and clinical situations, while assessing adherence to guidelines.

Our findings showed moderate to high agreement among European psychiatrists on pharmacological treatment for first-episode psychosis, cognitive and negative symptoms, prodromal symptoms, and pregnancy, with only moderate adherence to guidelines. Treatment preferences were similar for antipsychotic-induced metabolic side effects and treatment resistance, but guideline adherence in these areas was partial. Non-pharmacological interventions—such as psychotherapy, recovery-oriented care, cognitive remediation, and physical exercise—were generally underused, with exceptions for psychoeducation, lifestyle recommendations, and cognitive behavioral therapy in the prodromal phase.

These results highlight a gap between research evidence, guidelines, and real-world practice. Clinicians’ expertise and experience are key to shaping future guidelines in form of clinical consensus, particularly where practice diverges from recommendations.

**Disclosure of Interest:**

None Declared

